# Performance of Swarnadhara breeder hens supplemented with reduced levels of different copper forms

**DOI:** 10.14202/vetworld.2021.1371-1379

**Published:** 2021-05-29

**Authors:** Noor Aminullah, T. M. Prabhu, Jaya Naik, B. N. Suresh, H. C. Indresh

**Affiliations:** 1 Department of Animal Nutrition, Veterinary College, Karnataka Veterinary, Animal and Fisheries Sciences University, Bengaluru, Karnataka, India; 2 Department of Poultry Science, Veterinary College, Karnataka Veterinary, Animal and Fisheries Sciences University, Bengaluru, Karnataka, India; 3 Department of Livestock Farm Complex, Veterinary College, Karnataka Veterinary, Animal and Fisheries Sciences University, Hassan, Karnataka, India

**Keywords:** egg production, hatchability, nanoparticles, organic copper, poultry

## Abstract

**Background and Aim:**

Copper (Cu) is a vital mineral involved in various physiological and biochemical processes, growth, and productivity of animals and birds. Birds can absorb only a small fraction of Cu and most is excreted, contaminating soil and aquatic environment which is toxic for microorganisms, plants, animals, and humans. This study evaluated the possibility of use of organic and nanoparticles sources of Cu to reduce supplementation level without compromising the performance of breeder hens.

**Materials and Methods:**

A total of 224 Swarnadhara breeder hens were divided into seven treatment groups having four replicates in each. The basal diet (control) containing 20 ppm inorganic Cu (100% of standard recommendation) and six test diets containing 20, 15, and 10 ppm (100, 75, and 50% of standard recommendation) from Cu organic source, and 15, 10, and 5 ppm (75, 50, and 25%) from Cu nanoparticles (Cu-NP), were prepared and offered to respective treatment groups for a duration of 20 weeks.

**Results:**

The hen day egg production, hen housed egg production, feed conversion ratio egg mass, albumen index, yolk index, total fat content, and color score were not affected by the source and inclusion level of Cu. The feed intake was significantly (p<0.05) lower at 15 ppm and egg weight was significantly (p<0.05) higher at 10 ppm Cu-NP supplemental level, but was non-significant in other treatment groups compared to control. The body weight gain was significantly (p<0.05) higher at 20 ppm organic and 15 ppm Cu-NP inclusion. The egg shape index and Haugh unit were significantly (p<0.05) lower at 10 and 15 ppm of Cu-NP inclusion level, respectively. The shell thickness was improved (p<0.05) at 20 and 15 ppm organic and 15 and 10 ppm Cu-NP inclusion level. The egg fertility rate was shown to be significantly (p<0.05) higher at 20 ppm organic Cu inclusion group, but the hatchability based on total number of eggs set improved (p<0.05) at 20 and 15 ppm organic Cu inclusion level while all treatment groups were comparable to control. The hatchability of fertilized egg and chick’s quality significantly (p<0.05) improved, while embryonic and chick mortality after hatching before-sorting was significantly (p<0.05) reduced at 15 ppm of Cu-NP inclusion group.

**Conclusion:**

It was concluded that the inorganic Cu can be replaced with 50% of organic or 25% of nanoparticles form of Cu without jeopardizing the breeder hens’ productivity, egg quality characteristics, hatchability, and progeny.

## Introduction

The beneficial effect of copper (Cu) on the development and functioning of bird is well documented [1-3] and recognized to serve as a cofactor for many enzymes such as cytochrome oxidase, lysyl oxidase [4], ceruloplasmin, and superoxide dismutase [5]. Cu is essential for growth, production, reproduction, and health of bird. However, the major ingredients included in poultry feed unable to supply the required quantity of Cu and its deficiency is common in practical feeding [6]. Therefore, dietary supplementation of Cu is essential [7,8] to maintain the productivity of poultry. Further, the mineral’s absorption and utilization is reported to be poor [8,9] and a large amount of trace elements is excreted in the manure of livestock and poultry [10] and pollute the environment. Poultry manure applied on nitrogen basis in agricultural land contains 500% more Cu content than the plants requirement for optimum growth [8,11]. The excess Cu can lead to high environmental concentration which is toxic for many plants and grazing animals [12], affect the balance of other minerals [13] and encourage unfavorable microorganisms in soil and water sources [14]. Therefore, biotechnological interventions are made to increase the Cu bioavailability and absorption and reduce its environmental emission to avoid causing harmful effects on animal, poultry, and human health.

It is acknowledged that the organic form of Cu can improve its utilization and minimize its level in excreta and environmental pollution [15] due to its better absorption and metabolism properties [16]. Nollet *et al*. [15] reported no difference in performance between broilers fed high levels of inorganic Cu and low level of organic chelated Cu but significantly (p<0.05) lower fecal mineral excretion rates were observed in birds fed the organic form of Cu. Gheisari *et al*. [17] concluded that organic minerals (Cu, Mn, and Zn) at the dosage of 25-50% of inorganic minerals recommended level are sufficient to maintain performance and egg quality characteristics in laying hens.

Nanoparticle of Cu is another alternate form of Cu due to its novel properties such as their large surface-to-volume ratio [18], reactivity, and bioavailability [19] and prevents antagonism with other minerals [16]. Many studies have compared the availability of Cu nanoparticles (Cu-NP) to CuSO_4_ normal size when supplemented in animal diet, suggesting that the Cu-NP is better than its conventional inorganic form in enhancing the growth and performance of animals [20,21]. Kozłowski *et al*. [22] evaluated Cu-NP at three different levels (20, 10, and 2 mg/kg diet) to reduce its dietary supplemental level and concluded that Cu-NP can be reduced by 50% without compromising the growth rate and antioxidant status of turkey birds. In the previous studies, it was shown that Cu in organic and nanoparticles forms can enhance its bioavailability, growth performance, egg production, hatchability, immune-modulation, reduce pathogen load, and improve health of breeder hens [13].

Very limited work has been done yet to study the dietary lower level of Cu using organic and nanoparticles form on layer egg production, egg quality characteristics, hatchability, and progeny. The aim of this study was to find the most biologically effective form of Cu with higher efficiency and reduce supplementation level without compromising the performance of breeder hens.

## Materials and Methods

### Ethical approval

All the procedures with regard to the management and care of the birds followed during the trial were approved (letter no.: VCH/IAEC/2020/01) by the Institutional Animal Ethics Committee of the University (KVAFSU, Bidar, Karnataka).

### Study period and location

This experiment was conducted from July 2019 to November 2019 for a total of 20 weeks of production period at the Department of Poultry Science, Veterinary College, Hebbal, Bangalore, Karnataka, India, located at N 13.03°N and 77.60°E.

### Experimental birds and management

A genotype akin to country fowl named Swarnadhara breed was developed and released by Veterinary College, KVAFSU, Bengaluru, for rural scavenging conditions in the year 2007. A total of 224 Swarnadhara breeder hens of 28 weeks age at the Department of Poultry Science, Veterinary College, Bengaluru, were utilized for the study. All the birds were wing banded and randomly assigned to seven treatment groups with four replicates having eight hens and one cock in each (32 hens per treatment group). The birds were reared in a deep litter system with standard managemental practices till 48 weeks of age. All the breeders were exposed to 15 h light. The experiment was conducted from 29^th^ to 48^th^ week of the birds’ age, which constituted a total of 140 days and that was conveniently divided into five phases of 28 days each. All treatment groups received respective iso-nitrogenous and iso-caloric diets in all phases according to their production [23]. The daily required amount of feed was weighed and offered replicate-wise and recorded. Water was provided *ad libitum* during the trial period.

### Dietary treatments

The basal diet was formulated as per the recommendations of Indian Council of Agricultural Research (ICAR) [13] nutrient requirements except Cu for poultry. The Cu-NP were procured form M/s Matrix Nano Pvt Ltd., New Delhi, and inorganic Cu sulfate and organic Cu as Cu proteinate from M/s Zeus-Biotech Pvt. Ltd., Mysuru, Karnataka, India. The Cu-NP were prepared using induction coupled plasma (ICP) method having 98% purity and the particle size measured was 50-80 nm. The basal diet was supplemented with 20 ppm Cu from inorganic CuSO_4_ (T_1_) as this is the mainly used source of Cu in poultry feeding [9]. Treatment groups T_2_, T_3_, and T_4_ were supplemented with 20, 15, and 10 ppm levels (100, 75, and 50% of standard recommended level) of Cu from organic source as Cu proteinate, respectively. Treatment groups T_5_, T_6_, and T_7_ were supplemented at 15, 10, and 5 ppm levels (75, 50, and 25% of standard recommended level) of Cu-NP, respectively (Table-1). The ingredient composition of experimental basal diet is presented in Table-2 and its chemical composition in Table-3. The Cu content in the feed samples was estimated using Inductively Coupled Plasma - Optical Emission Spectrophotometer; Perkin Elmer Optima 8000. The Cu content of basal and experimental diets is presented in Table-3. The respective feed was weighed and offered daily to all experimental groups. Each bird received 150 g feed daily in a single dose during the experimental period from 29^th^ to 48^th^ week of age.

**Table-1:** Description of experimental groups.

Experimental group	Experimental diet	Cu supplementation level	

		Total Cu (ppm)	Compared to standard recommendation (%)
T_1_	Basal diet+ Inorganic Cu	20	100 (Control)
T_2_	Basal diet+ Organic Cu^1^	20	100
T_3_	Basal diet+ Organic Cu	15	75
T_4_	Basal diet+ Organic Cu	10	50
T_5_	Basal diet+ Cu-NP^2^	15	75
T_6_	Basal diet+ Cu-NP	10	50
T_7_	Basal diet+ Cu-NP	5	25

1 Copper proteinate,

2 Copper nanoparticles

**Table-2:** Ingredients composition of basal diet (quantity/100 kg).

Ingredients	Part
Yellow maize (kg)	58.86
Soybean meal (kg)	22.5
Deoiled rice bran (kg)	9.0
Oyster shell grit (kg)	4.5
Dicalcium phosphate (kg)	2.5
Mineral mixture-without copper* (kg)	2.12
Bacitracin methylene disalicylate (g)	70.0
Vitamin premix** (g)	15.0
Vitamin B complex*** (g)	25.0
DL-Methionine (g)	100.0
Hepatocare (g)	100.0
Common salt (g)	210.0
Total (kg)	100.0

* Contains: Ca 32%, P 9%, Fe 2000 ppm, Iodin 0.01%, Mn 0.4% and Zn 0.4%.

** Each gram contains: Vitamin A 82500 IU, Vitamin B2 50 mg, D3 12000 IU & K 10 mg.

*** Each gram contains: Vitamin B1 4 mg, Vitamin B6 8 mg, Vitamin B12 40 mg, Vitamin E 40 mg, Calcium D pantothenate 40 mg, Niacin 60 mg

**Table-3:** Chemical composition of the diet.

Nutrient	Composition	Total Cu content of experimental diets^a^	
ME (MJ/kg)^b^	11.67	Diet	mg/kg
Crude Protein (%)^a^	16.65	Basal diet*	8.03
Calcium (%)^a^	3.03	T_1_	28.04
Total phosphorus (%)^a^	0.77	T_2_	28.07
Lysine (%)^b^	0.78	T_3_	23.03
Methionine (%)^b^	0.41	T_4_	18.05
Selenium (ppm)^b^	0.25	T_5_	23.06
Zinc (ppm)^a^	86.51	T_6_	18.05
Fe (ppm)^a^	103.65	T_7_	13.04
Mn (ppm)^a^	59.55		
Vitamin A (IU/kg)^b^	14637.0		
Vitamin D3 (IU/kg)^b^	1846.00		
Vitamin E (IU/kg)^b^	91.37		
Thiamin (mg/kg)^b^	4.87		
Riboflavin (mg/kg)^b^	12.95		
Pyridoxine (mg/kg)^b^	6.25		
Cyanocoblamine (mg/kg)^b^	0.45		

Copper= Cu,

a Analyzed value,

b Calculated value,

* Without addition of supplemental Cu

### Production performance

The daily egg production was recorded replicate wise and hen day egg production (HDEP) and hen housed egg production (HHEP) were calculated on percent basis. The feed conversion ratio (FCR) was calculated on the basis of feed consumed per unit egg mass produced. Egg mass was calculated period wise considering number of eggs produced and egg weight. The bird initial and period wise body weight was recorded to evaluate the body weight gain during the experiment.

### Egg quality characteristics

Four eggs from each replicate were randomly selected on last day of each 28-day phase and evaluated for the external and internal quality characteristics. After the external characteristics study, each egg was broken and the entire contents were carefully emptied on a slab for metric measurement such as albumen index, yolk index, and Haugh unit score [24]. The egg yolk color was scored using matching technique Roche yolk color fan [25] and yolk fat content was evaluated as per AOAC [26] for the last phase of the experiment and expressed on dry matter basis.

### Fertility, hatchability, and progeny study

Egg produced during last 3 consecutive days of each phase were pooled and set replicate-wise for fertility, hatchability, and progeny study in incubator. The egg candled on day 18^th^ of incubation to sort and record fertile and infertile egg. Number of embryonic mortality, chick mortality after hatching-before sorting and live-good quality chicks were recorded on 21^st^ day of incubation. For progeny evaluation, four chicks per replicate (16 per treatment group) were randomly selected and subjected for quality evaluation using the method described by Tona *et al*. [27] with scoring based on activity, feathering and appearance, condition of eyes, conformation of legs, condition of navel area, remaining yolk sac, and status of the yolk membranes.

### Statistical analysis

The data were subjected to analysis of variance analysis using Statistical Package for the Social Sciences version16 (SPSS Inc., USA). The findings were subjected to test the H_o_ (Null hypothesis) at designed level of significance (p≤0.05). If the p≤0.05, value of traits was considered as a significant difference among the treatment groups. Mean comparison was made using Tukey *post hoc* test.

## Results

The egg production in terms of HDEP and HHEP, feed intake, FCR, body weight gain, egg weight, and egg mass is summarized in Table-4. The pooled data over the periods showed that different sources of Cu at different dietary inclusion level have no significant (p>0.05) effect on egg production, FCR, and egg mass of the hens. The feed intake was significantly (p<0.05) lower in T_5_ and egg weight was significantly (p<0.05) higher in T_6_ as compared to control (T_1_), while comparable with all other treatment groups. The weight gain was significantly (p<0.05) higher in T_2_ and T_5_ as compared to control but all other treatment groups were non-significant (p>0.05) with each other.

**Table-4:** Egg production, feed intake, feed conversion ratio, egg weight, egg mass and body weight gain influenced by different treatments.

Treatment group	Cu level (%) of requirement	Cu source	Hen day egg production (%)	Hen housed egg production (%)	Feed intake (g/day/hen)	FCR (kg feed intake/kg egg mass)	Egg weight (g)	Egg mass (g/day/hen)	Body weight gain (g)
T_1_	100	Inorganic	64.06± 1.66	63.06± 1.66	149.50± 0.10^b^	3.80± 0.07	58.26± 0.66^b^	39.27±0.59	50.13± 10.60^b^
T_2_	100	Organic	64.55± 1.33	64.55± 1.33	149.78± 0.05^b^	3.81± 0.06	59.04± 0.60^ab^	39.52± 0.65	99.63± 10.93^a^
T_3_	75	Organic	65.57± 1.55	63.82± 1.81	149.58± 0.13^b^	3.72± 0.05	59.99± 0.66^ab^	39.13± 0.84	70.41± 9.69^b^
T_4_	50	Organic	62.38± 1.60	62.39± 1.60	149.29± 0.06^b^	3.83± 0.06	60.03± 0.51^ab^	39.19± 0.74	77.56± 12.23^b^
T_5_	75	Nanoparticles	64.19± 1.45	63.97± 1.42	145.34± 0.56^a^	3.78± 0.09	60.03± 0.51^ab^	39.80± 0.88	96.31± 10.65^a^
T_6_	50	Nanoparticles	63.13± 1.52	63.13± 1.52	149.63± 0.07^b^	3.83± 0.07	60.84± 0.67^a^	39.38± 0.75	91.20± 10.14^b^
T_7_	25	Nanoparticles	64.38± 1.18	64.38± 1.18	149.59± 0.08	3.78± 0.06	59.65± 0.61^ab^	39.74± 0.65	78.44± 9.12^b^
SEM			3.546	3.631	0.462	0.168	2.881	1.760	71.375
p-value			0.824	0.959	<0.001	0.939	0.081	0.986	0.015

Mean values bearing different superscripts within the column differ significantly (p<0.05) , Cu=Copper, FCR=Feed conversion ratio

Egg external and internal quality characteristics, including shape index, albumen index, yolk index, yolk fat content, yolk color score, and shell thickness are presented in Table-5. The pooled data over the experimental periods indicated non-significant (p>0.05) variation in albumen index, yolk index, yolk fat content, and yolk color score in different treatment groups. The shape index was significantly (p<0.05) lower in T_6_ as compared to control, T_2_ and T_3_, while Haugh unit was significantly (p<0.05) lower in T_5_ compared to control and comparable with other treatment groups. The eggshell thickness was lesser in control, significantly (p<0.05) improved in T_2_, T_3_, and T_6_ as compared to control but highest (p<0.05) in T_5_ as compared to all treatment groups.

**Table-5:** Internal and external egg quality characteristics influenced by different treatments.

Treatment group	Cu level (%) of requirement	Cu source	Shape index	Albumen index	Yolk index	Haugh unit score	Shell thickness (mm)	Yolk fat content (%) dry matter basis	Yolk color score
T_1_	100	Inorganic	74.46± 0.52^b^	0.10± 0.00	0.44± 0.01	86.42± 0.56^a^	0.32± 0.00^a^	59.49± 0.99	7.28± 0.11
T_2_	100	Organic	75.27± 0.42^b^	0.10± 0.00	0.43± 0.01	84.02± 0.54^ab^	0.34± 0.00^b^	57.94± 1.89	7.33± 0.10
T_3_	75	Organic	74.46± 0.41^b^	0.09± 0.00	0.42± 0.01	84.41± 0.54^ab^	0.34± 0.00^b^	58.25± 1.54	7.31± 0.09
T_4_	50	Organic	73.65± 0.94^ab^	0.10± 0.00	0.43± 0.00	85.12± 0.56^ab^	0.33± 0.00^ab^	59.01± 0.60	7.17± 0.10
T_5_	75	Nanoparticles	73.19± 0.34^ab^	0.09± 0.00	0.43± 0.00	82.79± 0.58^b^	0.36± 0.00^c^	59.05± 0.89	7.16± 0.09
T_6_	50	Nanoparticles	70.35± 1.45^a^	0.10± 0.0	0.44± 0.01	85.96± 1.00^a^	0.34± 0.00^b^	59.78± 3.30	7.34± 0.98
T_7_	25	Nanoparticles	74.06±0.92^b^	0.09± 0.00	0.43± 0.01	85.20± 0.65^ab^	0.34± 0.00^ab^	59.57± 2.98	7.31± 0.93
SEM			3.869	0.017	0.024	3.128	0.017	2.136	0.483
p-value			<0.001	0.068	0.842	0.002	<0.001	0.993	0.792

Mean values bearing different superscripts within the column differ significantly (p<0.05) , Cu=Copper, FCR=Feed conversion ratio

The egg fertility and hatchability based on total and fertilized egg set, percentage of embryonic mortality and chick mortality after hatching-before sorting and chick’s quality are summarized in Table-6. The egg fertility was significantly (p<0.05) higher in T_2_ as compared to all other treatment groups except of T_3_. No significant effect of Cu-NP observed on egg fertility rate. The hatchability rate based on total number of eggs set was similar to that trend of fertility and no significant (p>0.05) difference was observed in treatment groups as compared to control. Hatchability percentage based on fertilized egg set was significantly (p<0.05) higher in T_3_, T_5_, and T_6_ as compared to control, but comparable with other treatment groups. Similarly, the chick’s and embryonic mortality rate was also significantly (p<0.05) lower in T_5_ and T_6_ as compared to T_4_ and T_7_. The chicks in T_5_ treatment group had significantly (p<0.05) higher Tona score as compared to control (T_1_), T_2_, T_3_, and T_4_, but comparable with T_6_ and T_7_.

**Table-6:** Fertility, hatchability, chick’s quality, and breeder hen survivability as influenced by different treatment.

Treatment group	Cu level (%) of requirement	Cu Source	Fertility (%)	Hatchability (%) based on total egg set	Hatchability (%) based on fertile egg set	Chick mortality (%) after hatching before sorting	Embryonic mortality (%)	Chick quality -Tona score (%)	Breeder hens’ survivability
T_1_	100	Inorganic	85.87± 0.83^acd^	74.90± 1.07^ab^	83.78± 1.02^a^	8.83± 1.04^a^	7.05± 0.95^ab^	89.50± 1.06^ad^	98.75± 0.86^ab^
T_2_	100	Organic	91.89± 0.84^b^	80.74± 1.23^a^	87.36± 1.00^acd^	5.62± 1.06^ab^	7.34± 0.90^ab^	92.45± 0.86^cd^	100.00± 0.00^b^
T_3_	75	Organic	89.00± 1.19^bc^	80.16± 1.22^a^	89.00± 1.30^cd^	4.47± 1.12^ab^	6.27± 0.80^ab^	92.12± 0.95^ac^	97.18± 1.15^a^
T_4_	50	Organic	85.03± 1.02^ac^	72.64± 1.44^b^	83.87± 0.92^a^	6.37± 1.21^ab^	7.93± 0.90^a^	88.60± 1.17^a^	100.00± 0.00^b^
T_5_	75	Nanoparticles	81.70± 0.97^a^	74.73± 1.51^b^	94.23± 1.29^b^	2.65± 0.84^b^	3.57± 0.91^b^	96.63± 0.96^b^	99.84± 0.15^b^
T_6_	50	Nanoparticles	83.25± 1.48^ad^	75.56± 1.72^ab^	92.09± 0.98^bd^	4.22± 0.88^b^	3.57± 0.91^b^	95.67± 0.69^bc^	100.00± 0.00^b^
T_7_	25	Nanoparticles	86.59± 1.30^dc^	75.70± 1.54^ab^	85.85± 1.23^ac^	5.37± 0.12^ab^	8.54± 1.00^a^	93.87± 0.87^bc^	100.00± 0.00^b^
SEM			2.667	3.352	2.674	2.562	2.191	4.327	1.308
p-value			<0.001	<0.001	<0.001	0.005	<0.001	<0.001	<0.001

Mean values bearing different superscripts within the column differ significantly (p<0.05) , Cu=Copper, FCR=Feed conversion ratio

## Discussion

### Production performance

In spite of reduced supplemental level of organic and nano-forms of Cu as compared to standard recommended level of normal form of inorganic CuSO_4_, the egg production, FCR, and egg mass are maintained without any adverse effect among different treatment groups as compared to control. Our study confirmed the better efficiency of organic [28] and nanoparticles [29] forms of Cu in egg production. The current result is in close agreement with the findings of Gheisari *et al*. [17] who illustrated that inclusion of organic minerals mixture (Zn, Mn, and Cu) at the level 50-75% less than NRC [30] recommendations is optimum to obtain optimal egg production, FCR, and egg mass. Saldanha *et al*. [31] reported that the organic minerals mixture (Zn, Mn, and Cu) or organic Cu [32] dietary supplementation level can be reduced by 30% without compromising the production performance of laying hens besides reducing Cu excretion to the environment. Our result also confirm with Ramesh [33] that Cu-NP dietary supplementation can be reduced by 75% as compared to its inorganic bulk form without compromising the egg production and egg mass. However, the findings are in contrast with Londero *et al*. [34] who concluded increased egg production due to addition of organic minerals in laying hens’ diets.

Significant (p<0.05) variation observed in respect of egg weight, feed intake, and body weight gain due to the source and inclusion level of Cu in different treatment groups. The egg weight was only significantly higher in T_6_ than in T_1_ group. The feed intake was significantly (P<0.05) lowest in T_5_ as compared to all other treatment groups while the FCR was comparable among all the groups. This reflects improved feed efficiency at 15 ppm inclusion level of Cu-NP in the diet as compared to 10 and 5 ppm but all other treatment groups were comparable. Similarly, Ramesh [33] demonstrated that Cu-NP dietary inclusion at 25-50% of requirement can reduce feed intake without compromising the FCR in laying hens. The lower feed intake without affecting FCR can be attributed to improved feed efficiency and energy utilization [29]. Cu plays a key role in cytochrome C oxidase enzyme which is essential for the production of adenosine triphosphate within the cell resulting in efficient energy utilization [35]. It is documented that the Cu-NP is crystalline in nature and has strong antimicrobial activity against both Gram-positive and Gram-negative bacteria [36], even showed the most efficient antimicrobial activity among the different forms of Cu [37,38]. This may be the reason for better gut health and improved nutrients utilization in T_5_ treatment group. In contrast, the organic form of Cu had no effect on feed intake as compared to control. The current result is in consistent with Yenice *et al*. [39] and Attia *et al*. [40] who reported that organic Cu form has no significant effect on feed consumption in laying hens.

The pooled data over the periods showed a significant (p<0.05) increase in egg weight at 50% supplemental level of Cu-NP (T_6_) as compared to control (T_1_), while comparable with all other treatment groups. The findings are in agreement with Attia *et al*. [40] who observed no significant effect of organic source of Cu on egg weight, but is contradicted with Lim and Paik [41] who found that organic form of Cu can increase the egg weight in laying hens. In the previous study, Ramesh [33] reported no significant increase of egg weight due to Cu-NP dietary supplementation in Vanaraja breeder hens, while our result showed significantly (p<0.05) higher egg weight at 10 ppm inclusion level. The 10 ppm of Cu-NP found to be the optimum level for egg weight. The better egg weight is attributed to higher fat content of egg yolk (59.78%) which is slightly higher compared to other treatment groups. Pesti and Bakalli [42] also suggested that Cu supplementation can affect reproductive physiology and lipid metabolism in layer bird.

The pooled values of body weight gain over the periods were significantly (p<0.05) different among treatment groups. The weight gain found to be dose-dependent response, but it could maintain the body weight at 50 and 25% inclusion level of organic and Cu-NP as compared to control, respectively. In the previous studies, it was reported that the growth performance can be maintained with 75% reduction of Cu-NP as compared with bulk form of CuSO_4_ supplemental level in growing chicks [43] and laying hens [33]. The result clearly demonstrates that dietary CuSO_4_ inorganic form can be replaced with 25% inclusion level of Cu-NP to maintain the body weight gain in laying hens. In contrast, it was reported that organic source and dose of minerals have no significant (p>0.05) effect on body weight changes in laying hens [39] and duck [44] as compared to inorganic form of CuSO_4_.

### Egg quality characteristics

The present study indicated significantly (p<0.05) lower egg shape index at 10 ppm Cu-NP supplemental level as compared to control while the reduced level of organic Cu could maintain the shape index. The findings are in agreement with Saleh *et al*. [45] and Yenice *et al*. [39] who reported no significant effect of organic minerals mixture on egg shape index.

Even at reduced supplemental levels of Cu from organic or nanoparticle sources, the egg albumen index, yolk index, yolk fat content, and yolk color Roche score were similar to that of control. The results corroborated with the findings of Saldanha *et al*. [31] who reported that dietary supplementation of organic minerals can be reduced up to 30% without compromising the egg quality parameters in semi-heavy layer hens. Attia *et al*. [40] also reported that no significant effect on egg yolk percentage, yolk index, albumen index, and shell thickness when 10, 60, and 120 ppm of organic Cu was used in dual-purpose breeding hens’ diets.

In the current experiment, the Haugh unit score was significantly (p<0.05) lower at 15 ppm Cu-NP inclusion level. However, the values in all other treatment groups are comparable to control. The findings are in agreement with Attia *et al*. [40] who noticed no significant difference in Haugh unit score of egg in laying hens fed diet supplemented with 10, 60, and 120 ppm organic Cu. Yenice *et al*. [39] and Stefanello *et al*. [28] also reported no effect of organic source of minerals on Haugh unit score in laying hens.

The eggshell thickness was significantly (p<0.05) highest at 15 ppm Cu-NP inclusion level and also improved at 20 and 15 ppm organic and at 10 ppm inclusion of Cu-NP as compared to control and other treatment groups. Cu plays role as cofactor of the lysyl-oxidase enzyme that is important in the formation of collagen cross-links present in the eggshell membranes [46]. Thus, the findings reinstate the improved bioavailability of both organic [13] and nano [19] forms of Cu over its coarse particles. Patra and Lalhriatpuii [29] reported that micro minerals nanoparticles can improve the eggshell quality in laying hens. Ramesh [33] also reported that Cu-NP at 50% reduction level can maintain eggshell quality. Our findings are also in consistent with Stefanello *et al*. [28] who reported that lower egg loss, higher shell thickness, and increased strength of shell from hens fed diet supplemented with organic source of minerals as compared to inorganic source. Carvalho *et al*. [32] also concluded that reduction of dietary organic minerals (Cu, Mn, and Zn) supplementation at 30% of inorganic source has comparable egg shell quality.

### Fertility, hatchability, and chick’s quality

The pooled data over the periods showed that the fertility rate is both dose - and form-dependent and affected particularly by Cu-NP. The fertility was significantly (p<0.05) higher in T_2 (_20 ppm organic Cu) group as compared to control and lower in T_5 (_15 ppm nano Cu) as compared to T_2_ and T_3_, but comparable with all other treatment groups. Studies indicated that feeding breeder hens with organic trace minerals can improve egg quality, fertility, and hatchability [47-49], and spermatozoa quality [34] which may be due to its greater bioavailability [50]. Our findings showed that 50 and 75% (10 and 15 ppm) supplemental level of organic Cu can maintain the egg fertility rate and significantly (p<0.05) improves it at 100% supplemental level as compared to standard recommendation of inorganic CuSO_4_. Our result is consistent with the findings of Attia *et al*. [44] who concluded that 10 ppm organic Cu is adequate for reproductive performance and egg quality in laying hens. The ICAR [23] recommended level for Cu is 20 ppm; therefore, the Cu supplementation from organic source can be reduced by 50% without affecting fertility and hatchability. The highest level of Cu-NP associated with the lowest fertility rate indicates that the Cu-NP can reduce the egg fertility rate. Very few reports are available in literature to demonstrate the effect of Cu-NP on egg fertility. However, Petruska *et al*. [51] and Sariozkan *et al*. [52] reported that nanoparticles can have spermatotoxic and/or oocyte toxic effects due to DNA fragmentation.

The hatchability rate based on total egg set was found to be better (p<0.05) in T_2_ and T_3_ as compared to T_4_ and T_5_ treatment groups, but all are comparable with control. The hatchability percentage based on fertilized egg set was significantly (p<0.05) affected by source and dose of Cu which was higher in T_3_, T_5_, and T_6_ as compared to control. Surprisingly, T_5_ with the lowest fertility rate (81.70%) is having significantly (p<0.05) highest (94.23%) hatchability among all treatment groups.

The significantly (p<0.05) lower chick mortality after hatching before-sorting in T_5_ and T_6_ dietary treatments as compared to control contributed for higher hatchability rate in T_5_. Similarly, the embryonic mortality rate was also significantly (p<0.05) lower in T_5_ and T_6_ as compared to T_4_ and T_7_. Therefore, losses of fertile egg were significantly reduced and hatchability improved at 15 ppm Cu-NP inclusion level. The hatchability was improved in all nanoparticles treatment groups (T_5_, T_6_, and T_7_) but significantly (p<0.05) highest in T_5_ as compared to control. The above findings indicate that dietary supplementation of Cu-NP can improve hatchability due to reduced embryonic mortality rate. The study revealed that Cu-NP at 25% inclusion level of CuSO_4_ normal form can maintain the hatchability and increased with incremental Cu level. Yenice *et al*. [39] reported that organic minerals had no significant effect on total hatchability and fertility but can significantly (p<0.05) improve hatchability of fertilized egg. In the current study, the organic Cu improved the fertility, hatchability, and progenies quality as well. Similar findings were reported by Saber *et al*. [53] who observed improved hatching and progenies performance when inorganic minerals mixture was replaced with 50% level of organic source in broiler breeder hens.

The chick quality evaluation score was better with increased supplemental dose of organic or nanoparticle of Cu. All the groups supplemented with nanoparticles (T_5_, T_6_, and T_7_) scored significantly (p<0.05) higher values as compared to control. The data showed that the chick quality can be supported at 50 and 75% reduced level of organic and nanoparticles forms of Cu compared to inorganic CuSO_4_, respectively, and can improve with higher supplemental level. The improved hatchability and chick quality are the result of novel properties of Cu-NP such as antimicrobial effects [9] and its important role in enzyme (cytochrome oxidase) that are vital in the cellular energy generation [4]. It is hypothesized that Cu-NP can affect the metabolic rate, thereby increasing oxygen (O_2_) consumption, improve embryogenesis, hatchability, and consequently the chick growth rate [54]. It was reported that Cu-NP, especially during egg incubation period can stimulate the metabolic rate of broiler embryos to a greater level than do bulk form of CuSO_4_. This could be associated with that Cu-NP has stronger bioactivity affecting the blood vessel formation and growth to a greater degree than CuSO_4_ [20]. The survivability rate of hens was affected in T_3_, but comparable with control group.

## Conclusion

It was concluded that the supplemental Cu level (20 ppm) in the form of inorganic CuSO_4_ in the diets can be reduced to 50 and 25 % when Cu supplemented in organic (10 ppm) and nanoparticle forms (5 ppm), respectively, without any adverse effect on productive performance, egg quality characteristics, and hatchability of Swarnadhara breeder hens. The egg fertility, shell thickness, and body weight gain were better at 20 ppm Cu inclusion in organic form. The Cu-NP has better effect in respect of FCR, egg weight, and shell thickness at 15 and 10 ppm inclusion level. The egg fertility rate was not affected by treatment diets than control, while hatchability of fertilized egg and progeny quality improved. Future studies should aim to assess Cu retention in the body tissues or egg to ascertain the bioavailability of Cu from different Cu sources or forms.

## Authors’ Contributions

TMP: Conceived and designed the research. AN: Performed the experiment and nalyzed the data. TMP, AN, JN, BNS, and HCI: Interpreted the data. AN, JN, BNS, and HCI: Drafted the manuscript. TMP: Edited the manuscript. All authors read and approved the final manuscript.
